# Activation of Endoplasmic Reticulum Stress in Granulosa Cells from Patients with Polycystic Ovary Syndrome Contributes to Ovarian Fibrosis

**DOI:** 10.1038/s41598-017-11252-7

**Published:** 2017-09-07

**Authors:** Nozomi Takahashi, Miyuki Harada, Yasushi Hirota, Emi Nose, Jerilee MK Azhary, Hiroshi Koike, Chisato Kunitomi, Osamu Yoshino, Gentaro Izumi, Tetsuya Hirata, Kaori Koga, Osamu Wada-Hiraike, R. Jeffrey Chang, Shunichi Shimasaki, Tomoyuki Fujii, Yutaka Osuga

**Affiliations:** 1 Department of Obstetrics and Gynecology, Faculty of Medicine, The University of Tokyo, 7-3-1, Hongo, Bunkyo, Tokyo 113-8655 Japan; 2 Department of Obstetrics and Gynecology, Faculty of Medicine, University of Toyama, 2630 Sugitani, Toyama, 930-0194 Japan; 3 Department of Reproductive Medicine, University of California San Diego, 9500 Gilman Drive, La Jolla, CA 92093-0633 USA

## Abstract

Recent studies report the involvement of intra-ovarian factors, such as inflammation and oxidative stress, in the pathophysiology of polycystic ovary syndrome (PCOS), the most common endocrine disorder of reproductive age women. Endoplasmic reticulum (ER) stress is a local factor that affects various cellular events during a broad spectrum of physiological and pathological conditions. It may also be an important determinant of pro-fibrotic remodeling during tissue fibrosis. In the present study, we showed that ER stress was activated in granulosa cells of PCOS patients as well as in a well-established PCOS mouse model. Pharmacological inducers of ER stress, tunicamycin and thapsigargin, were found to increase the expression of pro-fibrotic growth factors, including transforming growth factor (TGF)-β1, in human granulosa cells, and their expression also increased in granulosa cells of PCOS patients. By contrast, treatment of PCOS mice with an ER stress inhibitor, tauroursodeoxycholic acid or BGP-15, decreased interstitial fibrosis and collagen deposition in ovaries, accompanied by a reduction in TGF-β1 expression in granulosa cells. These findings suggest that ER stress in granulosa cells of women with PCOS contributes to the induction of pro-fibrotic growth factors during ovarian fibrosis, and that ER stress may serve as a therapeutic target in PCOS.

## Introduction

Polycystic ovary syndrome (PCOS), the most common endocrine disorder among reproductive age women, affects 6−10% of them, and it is the most common cause of anovulatory infertility^1^. The pathophysiology of PCOS is complex and reflects the interactions between various factors including disordered gonadotropin secretion, androgen excess, insulin resistance, ovarian dysfunction, and follicular arrest^2^. Despite its unclear pathophysiology, recent studies reveal that intra-ovarian factors, such as chronic inflammation and oxidative stress, play a crucial role in the pathogenesis of PCOS^2–9^.

PCOS is characterized by a thickening of the ovarian capsule and stroma due to increased collagen deposition and fibrous tissue^10^. Since fibrotic conditions in different organs have different etiologies, the underlying mechanism by which fibrosis is induced in PCOS is not clear. Recent studies reveal the essential roles of transforming growth factor (TGF)-β1 and connective tissue growth factor (CTGF), a downstream factor mediating the pro-fibrotic actions of TGF-β1, in granulosa cells during extracellular matrix remodeling in the ovary^11–14^. Consistent with this notion, a higher serum level and an increased ovarian expression of TGF-β1 in PCOS patients supports the involvement of TGF-β1 in the pathogenesis of PCOS^14, 15^, although neither the mechanism regulating TGF-β1 expression nor the role of the upregulated TGF-β1 level in the pathogenesis of PCOS has been elucidated.

Endoplasmic reticulum (ER) stress is a local factor that is closely related to inflammation and oxidative stress. ER stress, which involves the accumulation of unfolded or misfolded proteins in the ER, is caused by various physiological and pathological conditions, including oxidative stress and inflammation, which increase the demand for protein folding or attenuate the protein-folding capacity in the ER^16–20^. ER stress results in the activation of several signal transduction cascades collectively termed the unfolded protein response (UPR), which affects a wide variety of cellular functions^21^. Recently, it has been recognized that ER stress and UPR are important determinants of pro-fibrotic remodeling in tissue fibrosis^22, 23^. For example, ER stress, activated by the hepatitis C virus (HCV) in HCV hepatitis or the accumulation of mutated alpha-1 antitrypsin in genetic liver disease, was shown to upregulate the release of the pro-fibrotic growth factor, TGF-β1, from hepatocytes and to induce liver fibrosis^24, 25^. ER stress also exerts its pro-fibrotic role in angiotensin II-induced hypertension by increasing the TGF-β1 level in the aorta and heart, resulting in vascular endothelial dysfunction and cardiac damage^26^.

We previously demonstrated that ER stress is activated in granulosa cells during follicular growth, and that activated ER stress in granulosa cells influences the regulation of cellular functions^27–29^. ER stress stimulates the production of vascular endothelial growth factor A by granulosa cells^28^, whereas it inhibits the production of progesterone by human chorionic gonadotropin by these cells^29^. These findings suggest that ER stress and UPR are important regulators of physiological events in granulosa cells. Based on these findings, we hypothesize that ER stress is activated in granulosa cells of PCOS patients, and that activated ER stress induces ovarian fibrosis by modulating pro-fibrotic growth factor expression in granulosa cells. To test this hypothesis, we first examined the activation of ER stress using granulosa cells from PCOS patients, the ovaries from PCOS patients, and the well-established dehydroepiandorosterone (DHEA)-treated PCOS mouse model, and then determined the *in vitro* effects of ER stress on TGF-β1 production in cultured human granulosa cells. We also examined the *in vivo* effects of ER stress inhibitors, tauroursodeoxycholic acid (TUDCA) and BGP-15, on ovarian fibrosis in the PCOS mouse model. We decided to utilize TUDCA and BGP-15 as ER stress inhibitors in this study because they are safe in humans, accordingly they may be therapeutic agents by targeting activated ER stress. TUDCA has been used to treat liver diseases and dissolve gallstones^30^ and recent studies reveal that TUDCA functions as a chemical chaperone that attenuates protein misfolding and reduces ER stress^31^. On the other hand, BGP-15 has been shown to be safe and well tolerated in phase 2 clinical trials for diabetes and insulin resistance^32^. It is a hydroxylamine derivative that amplifies the endogenous stress response by altering the organization of cholesterol-rich membrane domains to specifically target stressed cells and a pharmacological co-inducer of the chaperone HSP72^32, 33^.

## Results

### ER stress is activated and the expression of TGF-β1 is increased in granulosa cells of PCOS patients

**Figure 1:**
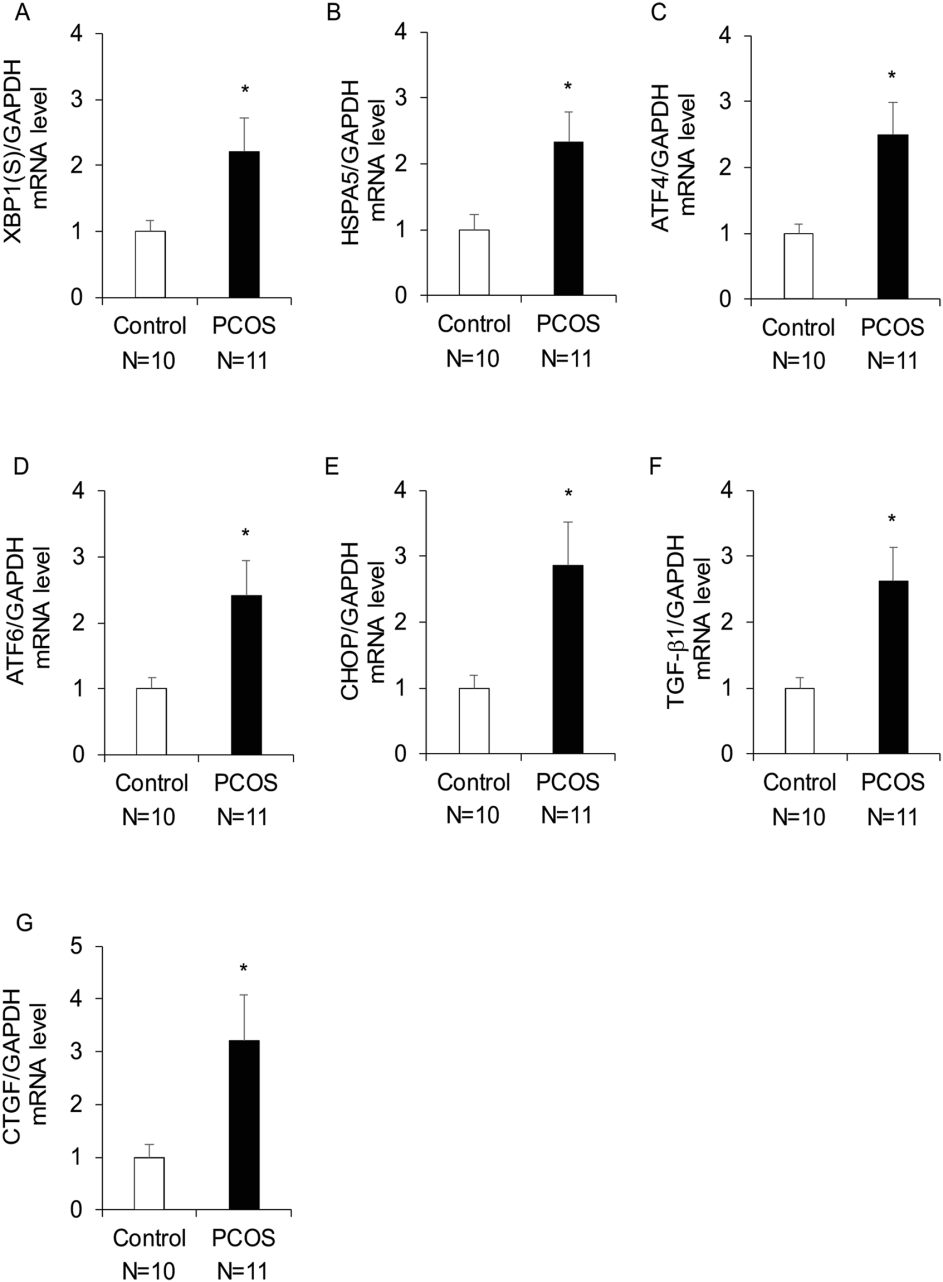
The expression of UPR genes and pro-fibrotic growth factors in granulosa-lutein cells from control (n = 10) and PCOS (n = 11) patients. The expression levels of (**A**–**E**) UPR genes, XBP1(S), HSPA5, ATF4, ATF6, and CHOP mRNA and (**F**,**G**) pro-fibrotic growth factors, TGF-β1 and CTGF mRNA in granulosa-lutein cells were measured by real-time PCR and normalized to that of GAPDH. Increased UPR gene expression was indicative of ER stress activation. The values represent means ± SEM. *p < 0.05.

To examine the activation of ER stress, the mRNA expression levels of various UPR genes were measured in granulosa-lutein cells from PCOS (n = 11) and control (n = 10) patients. There were no significant differences in terms of age, body mass index (BMI), the number of oocytes retrieved, and the serum levels of follicle stimulating hormone (FSH), estradiol (E2), and prolactin (PRL) on days 3−5 of the menstrual period between the groups. However, serum luteinizing hormone (LH), the LH/FSH ratio, and anti-Müllerian hormone (AMH) levels were significantly higher in PCOS patients than in control patients (Supplementary Table 1). The levels of the spliced form of X-box-binding protein 1 [XBP1(S)], heat shock 70 kDa protein 5 (HSPA5), activating transcription factor 4 (ATF4), ATF6, and C/EBP homologous protein (CHOP) were examined as markers of ER stress activation. As shown in Fig. 1A–E, XBP1(S), HSPA5, ATF4, ATF6, and CHOP mRNA expression levels were significantly higher in granulosa-lutein cells of PCOS patients than in control patients. The expression of the pro-fibrotic cytokine TGF-β1, and its downstream factor CTGF, was examined in human granulosa-lutein cells obtained from PCOS and control patients, which were the same samples as used for the examination of the activation of ER stress. As shown in Fig. 1F,G, TGF-β1 and CTGF mRNA expression levels were significantly higher in granulosa-lutein cells of PCOS patients than in control patients.

**Figure 2:**
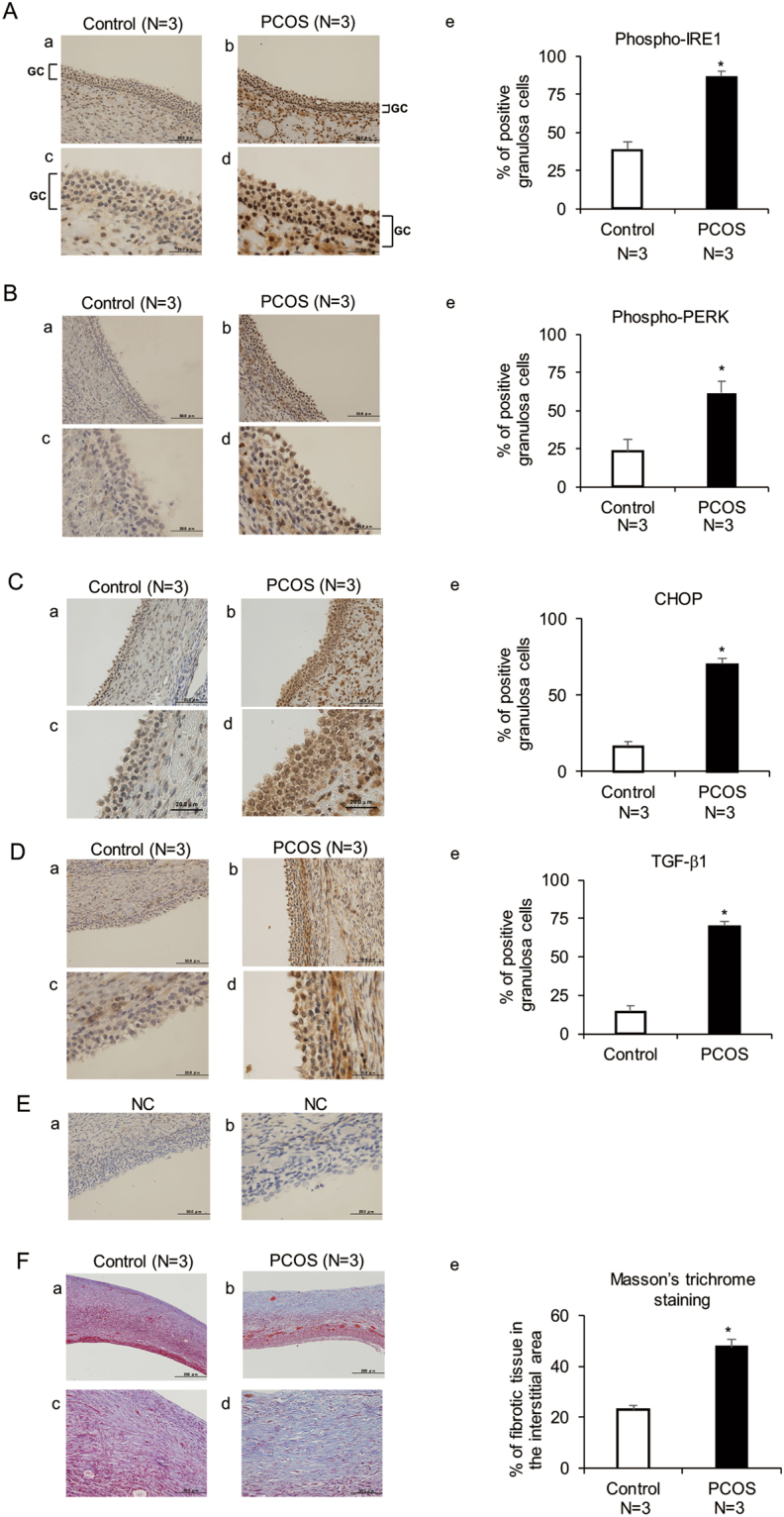
Phospho-IRE1, phospho-PERK, CHOP, and TGF-β1 protein expression levels and the area of fibrotic tissue in the ovaries of control and PCOS patients. Immunohistochemical analysis was performed on the ovaries of 3 PCOS and 3 control patients. (**A**–**D**) Cross-sections of ovaries were stained with an anti-phospho-IRE1, anti-phospho-PERK, anti-CHOP, or anti-TGF-β1 antibody, counterstained with hematoxylin. (**E**) Controls for background level stained with isotype IgG and hematoxylin. (**F**) Cross-sections of ovaries were stained with Masson’s trichrome stain. Fibrotic tissue was stained blue. (**A**–**D**,**F**) (a–d) show the representative sections. Lower panels (c,d) show highly magnified views corresponding to (a,b). (e) show the quantitative analysis of (**A**–**D**) immunohistochemical staining and (**F**) Masson’s trichrome staining. (**E**) A right panel (b) shows a highly magnified view corresponding to a left panel (a). The scale bars in (**A**–**D**) (a,b) and (**E**) (a) indicate 50 μm, while those in (**A**–**D**) (c,d) and (**E**) (b) indicate 20 μm. The scale bars in (**F**) (a,b) and (**F**) (c,d) indicate 200 μm and 50 μm, respectively. *p < 0.05. GC, granulosa cell layer; NC, negative control.

To confirm the activation of ER stress and the increased expression of TGF-β1 in granulosa cells of PCOS patients, immunohistochemical analysis was performed to examine the distribution of activated ER stress sensor proteins, inositol-requiring enzyme 1 (IRE1) and double-stranded RNA-activated protein kinase-like ER kinase (PERK), and CHOP, as well as TGF-β1, in the ovaries of PCOS patients. Upon activation, IRE1 and PERK undergo phosphorylation, thus triggering UPR. As shown in Fig. 2A–D, phospho-IRE1, phospho-PERK, and CHOP immunoreactivity was increased in granulosa cells of PCOS patients compared with that of the control group, accompanied with an increase in TGF-β1 immunoreactivity in these cells. In addition, as shown in Fig. 2F, Masson’s trichrome staining revealed that fibrotic tissue in the interstitial area was increased in PCOS patients compared with that in the control group.

### ER stress is activated and the expression of TGF-β1 is increased in granulosa cells from PCOS mice

**Figure 3:**
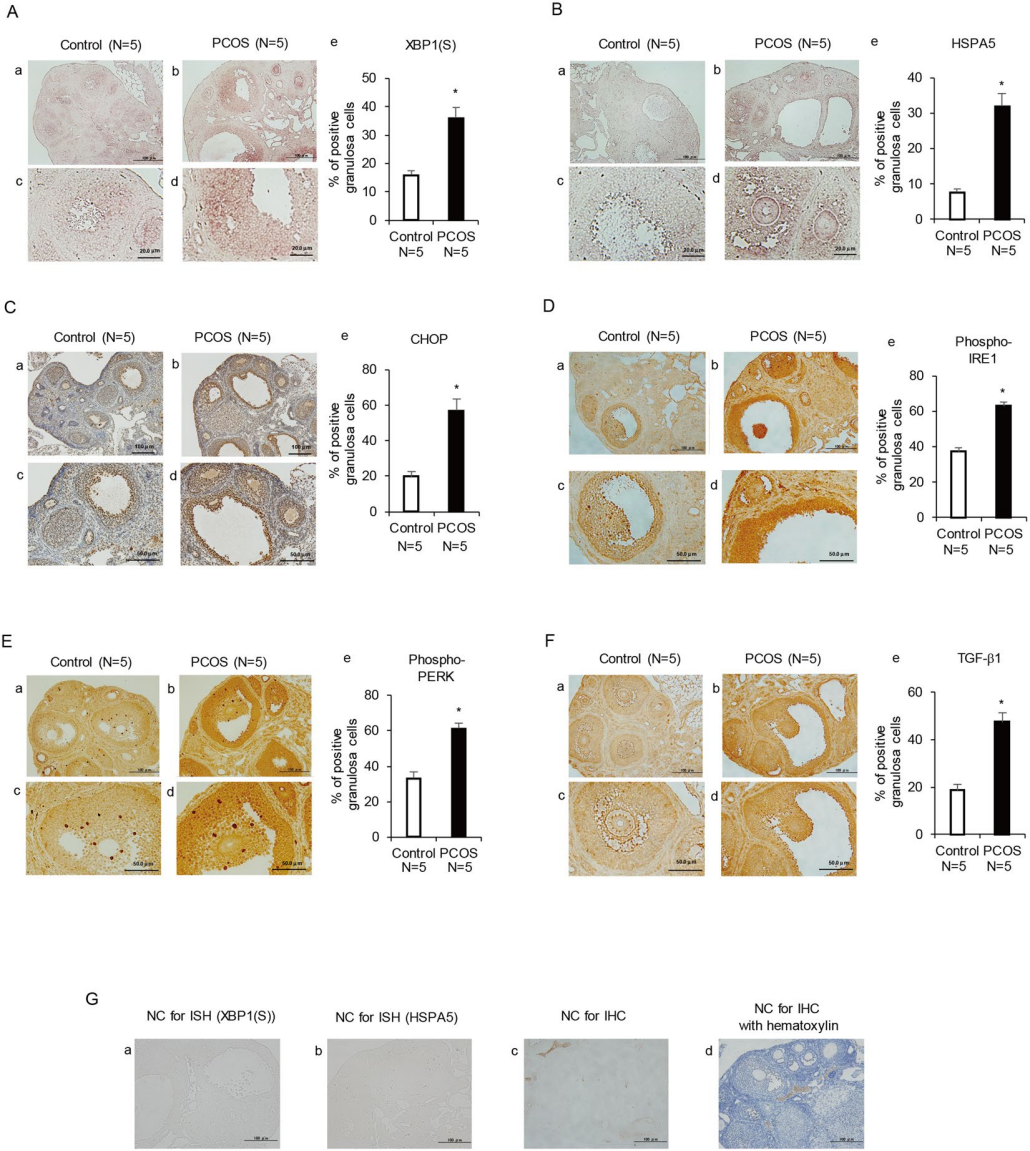
XBP1(S) and HSPA5 mRNA expression levels, and CHOP, phospho-IRE1, phospho-PERK, and TGF-β1 protein expression levels in ovaries of control and PCOS mice. Three-week-old female mice were divided into two groups. The control group (n = 5) was s.c. injected daily with sesame oil for 20 days. The PCOS group (n = 5) was s.c. injected daily with DHEA (6 mg/100 g of body weight) for 20 days. The ovaries were collected on day 21. (**A**,**B**) Cross-sections of ovaries from control and PCOS mice were hybridized with a DIG-labeled antisense XBP1(S) or HSPA5 probe. (**C**–**F**) Cross-sections of ovaries from control and PCOS mice were stained with an anti-CHOP antibody counterstained with hematoxylin, or an anti-phospho-IRE1, anti-phospho-PERK, or anti-TGF-β1 antibody. (**G**) Controls for background level (a,b) hybridized with sense probe, (c) stained with isotype IgG, and (d) stained with isotype IgG and hematoxylin. (**A**–**F**) (a–d) show the representative sections. Lower panels (c,d) show highly magnified views corresponding to (a,b). (e) show the quantitative analysis of (**A**,**B**) *in situ* hybridization and (**C**–**F**) immunohistochemical staining. The scale bars in (**A**–**F**) (a,b) and (**G**) (a–d) indicate 100 μm. The scale bars in (**A**,**B**) (c,d) and (**C**–**F**) (c,d) indicate 20 μm and 50 μm, respectively. *p < 0.05. NC, negative control; ISH, *in situ* hybridization; IHC, immunohistochemistry.

We then examined the activation of ER stress and the expression of TGF-β1 in the ovaries of PCOS mice. The serum testosterone level was higher and the number of cystic follicles in the ovary was larger in PCOS mice than in control mice. PCOS mice exhibited acyclicity with prolonged estrous cycle, consistent with earlier studies (Supplementary Figure 1)^34–36^. *In situ* hybridization analysis revealed that the expression of UPR genes, namely, XBP1(S) and HSPA5, was increased in granulosa cells of PCOS mice compared with that of the control group (Fig. 3A,B). To confirm the activation of ER stress, immunohistochemical analysis was performed to examine the distribution of CHOP and the activated ER stress sensor proteins, IRE1 and PERK, in granulosa cells of PCOS mice. As shown in Fig. 2C–E, immunoreactivity of CHOP, phospho-IRE1, and phospho-PERK was increased in granulosa cells of PCOS mice compared with that of the control group. Figure 2F shows the increased immunoreactivity of TGF-β1 in granulosa cells of PCOS mice compared with that of the control group. These observations in PCOS mice are in accordance with those in human specimens.

### ER stress induces TGF-β1 and CTGF expression in cultured human granulosa-lutein cells

**Figure 4:**
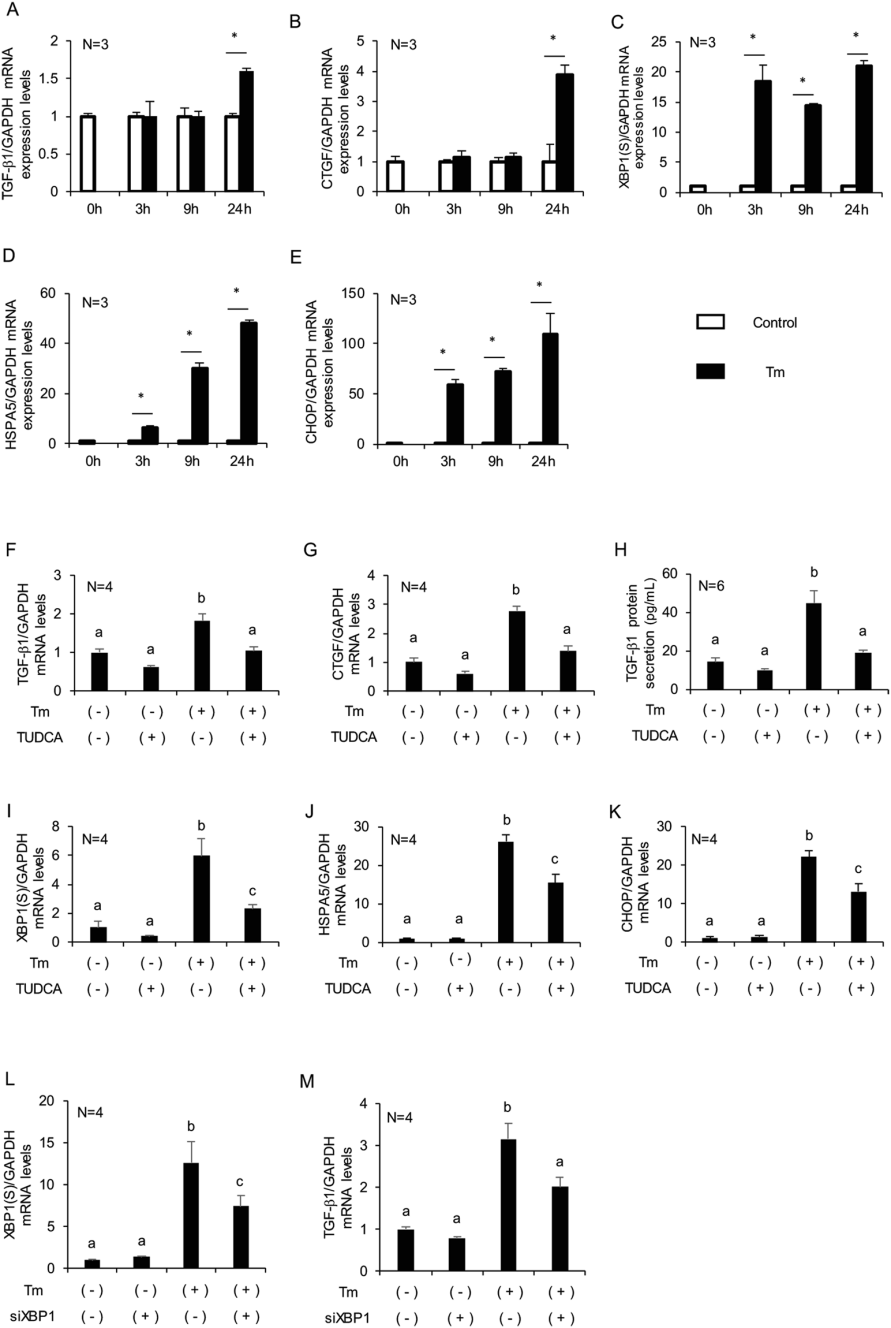
Effects of ER stress on TGF-β1 and CTGF mRNA expression levels and TGF-β1 secretion by cultured human granulosa-lutein cells. (**A**–**E**) Granulosa-lutein cells were treated with tunicamycin at 2.5 μg/mL for 0, 3, 9, or 24 h. (**F**–**K**) Granulosa-lutein cells were pre-incubated with TUDCA at 1 mg/mL for 24 h, followed by treatment with tunicamycin at 2.5 μg/mL for 24 h. (**L,M**) Granulosa-lutein cells were transfected with siRNA (50 nM) or negative control siRNA (50 nM) for 24 h and then, treated with tunicamycin (2.5 μg/mL) for 24 h. (**A**,**F**,**M**) TGF-β1, (**B**,**G**) CTGF, (**C,I,L**) XBP1(S), (**D**,**J**) HSPA5, and (**E**,**K**) CHOP mRNA expression levels in granulosa-lutein cells were measured by real-time PCR and normalized to that of GAPDH. The values represent means ± SEM of triplicate or quadruplicate experiments, relative to the mean control value. (**H**) The bioactive TGF-β1 level in cultured cell supernatants was measured by ELISA. The values represent means ± SEM of sextuplicate experiments. The results are representative of at least three independent experiments using three different samples. The letters denote significant differences between groups. *p < 0.05. Tm, tunicamycin; TUDCA, tauroursodeoxycholic acid.

To confirm the effects of ER stress on TGF-β1 expression in granulosa cells, cultured human granulosa-lutein cells were treated with tunicamycin, an ER stress inducer. As shown in Fig. 4A,B, tunicamycin treatment increased TGF-β1 and CTGF mRNA expression in granulosa-lutein cells compared with that of the control group, following an increase in UPR genes, XBP1(S), HSPA5, and CHOP mRNA (Fig. 4C–E). Pretreatment with the ER stress inhibitor TUDCA abrogated the effects of tunicamycin on the expression of TGF-β1 and CTGF (Fig. 4F,G), with a concomitant reduction in XBP1(S), HSPA5, and CHOP mRNA expression (Fig. 4I–K). For further confirmation, we examined the effect of treatment with thapsigargin, another ER stress inducer, and obtained similar results (Supplementary Figure 2). By ELISA, we found that tunicamycin induced bioactive TGF-β1 secretion by granulosa-lutein cells, which was abrogated by pretreatment with TUDCA (Fig. 4H). Next, to examine the molecular mechanisms implicated in the upregulation of TGF-β1 production by tunicamycin, we knocked down mRNA of XBP1(S), a transcription factor activated by ER stress, by RNA interference. XBP1(S) expression was knocked down by 41%, and TGF-β1 mRNA expression was reduced 36% (Fig. 4L,M).

### ER stress inhibitors attenuate ovarian fibrosis in PCOS mice, with a concomitant reduction in the expression of pro-fibrotic growth factors

**Figure 5:**
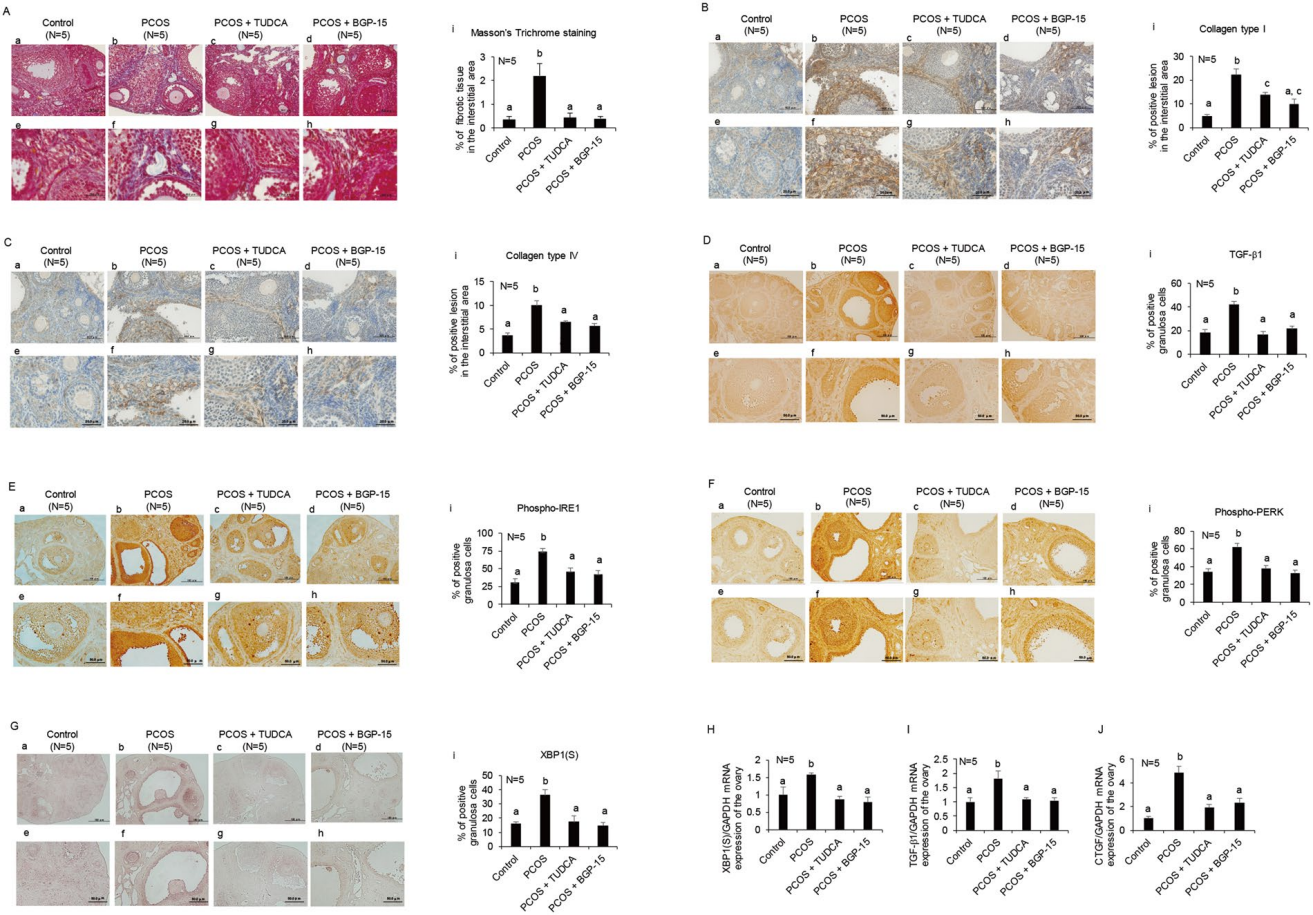
Effects of ER stress inhibitors on ovarian fibrosis in PCOS mice. Three-week-old female mice were divided into four groups. The control group (n = 5) was s.c. injected daily with sesame oil, followed by the oral administration of saline for 20 days. The PCOS group (n = 5) was s.c. injected daily with DHEA (6 mg/100 g of body weight), followed by the oral administration of saline for 20 days. The PCOS + TUDCA group (n = 5) was s.c. injected daily with DHEA, followed by the oral administration of TUDCA (50 mg/100 g of body weight) for 20 days. The PCOS + BGP-15 group (n = 5) was s.c. injected daily with DHEA, followed by the oral administration of BGP-15 (3 mg/100 g of body weight) for 20 days. The ovaries were collected on day 21. (**A**) Cross-sections of ovaries were stained with Masson’s trichrome stain. Fibrotic tissue was stained blue. (**B**–**F**) Cross-sections of ovaries were stained with collagen type I, collagen type IV, TGF-β1, phospho-IRE1, and phospho-PERK. (**G**) Cross-sections of ovaries were hybridized with a DIG-labeled antisense XBP1(S) probe. (**A**–**G**) (a–h) show the representative sections. Lower panels (e–h) show highly magnified views corresponding to (a–d). (i) show the quantitative analysis of (**A**) Masson’s trichrome staining, (**B**–**F**) immunohistochemical staining, and (**G**) *in situ* hybridization. The scale bars in (**A**–**C**) (a–d) and (**A**–**C**) (e–h) indicate 50 μm and 20 μm, respectively. The scale bars in (**D**–**G**) (a–d) and (**D**–**G**) (e–h) indicate 100 μm and 50 μm, respectively. (**H**–**J**) XBP1(S), TGF-β1, and CTGF mRNA expression levels in mouse ovaries were measured by real-time PCR and normalized to that of GAPDH. The letters denote significant differences between groups. TUDCA, tauroursodeoxycholic acid.

The *in vivo* effects of the ER stress inhibitor on ovarian fibrosis were tested using TUDCA, which was orally administrated to PCOS mice. To confirm the results, we also utilized another ER stress inhibitor, BGP-15. As shown in Fig. 5A, Masson’s trichrome staining revealed that fibrotic tissue in the interstitial area was increased in PCOS mice compared with that in the control group. In addition, the levels of collagen type I in the interstitial area and collagen type IV in the basement membrane were higher in the ovaries of PCOS mice than in those of control mice (Fig. 5B,C). The administration of TUDCA or BGP-15 attenuated the increase in the area of fibrotic tissue and the expression of collagen type I and IV in PCOS mice (Fig. 5A–C). Furthermore, TGF-β1 protein expression in the ovaries of PCOS mice was increased in granulosa cells compared with that of the control group. The administration of TUDCA or BGP-15 decreased TGF-β1 protein expression in granulosa cells of PCOS mice (Fig. 5D). As shown in Fig. 5E–G, phospho-IRE1 and phospho-PERK protein expression and XBP1(S) mRNA expression in granulosa cells of PCOS mice was reduced by treatment with TUDCA or BGP-15, suggesting that administration of these inhibitors decreased ER stress in granulosa cells of PCOS mice. Quantitative PCR analysis demonstrated that the increased mRNA expression of XBP1(S), TGF-β1, and CTGF in the ovaries of PCOS mice was inhibited by the administration of TUDCA or BGP-15 (Fig. 5H–J), consistent with the histological data. TUDCA and BGP-15 administration did not improve the estrous cyclicity and the cystic follicle counts of PCOS mice (Supplementary Figure 3).

## Discussion

Our results show that ER stress was activated in granulosa cells of both PCOS patients and the DHEA-treated PCOS mouse model. ER stress stimulated the expression of pro-fibrotic growth factors, including TGF-β1 in human granulosa cells. These growth factors were also increased in granulosa cells of PCOS patients. In the PCOS mouse model, treatment with ER stress inhibitors decreased interstitial fibrosis and collagen deposition in the ovary, which was accompanied by a reduction in TGF-β1 expression in granulosa cells.

In granulosa cells of PCOS patients, the expression of the UPR genes, XBP1(S), HSPA5, ATF4, ATF6, and CHOP, was upregulated. The UPR is a group of signal transduction cascades that are activated by ER stress^16, 17^. The increased expression of UPR genes is indicative of ER stress activation in granulosa cells of PCOS patients. The activation of ER stress is sensed by ER stress sensor proteins, such as IRE1 and PERK, which are phosphorylated upon activation, followed by the induction of the UPR. XBP1(S) expression is induced by the activation of IRE1, and CHOP is induced by the activation of PERK, while HSPA5 lies downstream of both IRE1 and PERK^16, 21^. The histological findings in human ovaries, which showed an increase in the immunoreactivity of phospho-IRE1, phospho-PERK, and CHOP in granulosa cells of PCOS patients, confirm the activation of ER stress in these cells. For further confirmation of the activation of ER stress in PCOS granulosa cells, we utilized a PCOS mouse model. *In situ* hybridization data showed an increase in XBP1 (S) and HSPA5 mRNA expression in granulosa cells of PCOS mice and immunohistochemical analysis revealed an increase in the immunoreactivity of CHOP, phospho-IRE1, and phospho-PERK in granulosa cells of PCOS mice, consistent with the findings in human granulosa cells of PCOS patients. ER stress is induced by a broad spectrum of physiological and pathological conditions, including oxidative stress and inflammation, that increase the demand for protein folding or attenuate the protein-folding capacity in the ER^16–20^. In turn, ER stress acts as an inducer of oxidative stress and inflammation. Additionally, inflammation is also induced by oxidative stress^19, 37–39^^,^. Thus, ER stress, oxidative stress, and inflammation are closely related to each other. Recent studies show that the inflammatory status in granulosa cells, as well as low grade systemic inflammation, is a key feature of the pathophysiology of PCOS^2–6^. Moreover, androgen excess, another key feature of the PCOS pathophysiology, is closely related to local inflammation. Androgen production by theca-interstitial cells is stimulated by inflammatory stimuli and, in turn, the treatment of granulosa cells with androgens induces the production of pro-inflammatory cytokines^6, 40^. Higher levels of oxidative stress markers in follicular fluid and granulosa cells of PCOS patients indicate that local oxidative stress may also contribute to the pathogenesis of PCOS^5–9^. It is plausible that ER stress, oxidative stress, and inflammation in granulosa cells cooperatively exacerbate the follicular microenvironment of the PCOS ovary. Additionally, insulin resistance, another key feature of the PCOS pathophysiology, might be implicated in the activation of ER stress in granulosa cells of PCOS patients, either directly or indirectly. Given that glucotoxic insults directly activate ER stress in adipocytes, which mediates induction of an inflammatory phenotype in these cells^41^, insulin resistance might directly activate ER stress in granulosa cells. In addition, insulin resistance might indirectly activate ER stress in granulosa cells, by inducing systemic or local inflammation. Insulin resistance causes alteration of the characteristics of adipocytes, resulting in systemic inflammation. Alternatively, insulin resistance induces local inflammation by increasing local androgen concentrations, since insulin stimulates ovarian androgen production and reduces hepatic sex hormone binding globulin synthesis, thereby contributing to hyperandrogenism^2^. In a previous study, we demonstrated that ER stress activated in the follicles of obese women, together with inflammation and oxidative stress, interferes with progesterone production^29^. Interestingly, the patients included in the present study were non-obese PCOS patients of Asian ethnicity, and the DHEA-induced PCOS mouse model utilized in the present study also exhibited minimal changes in body weight, which is consistent with the well-characterized phenotype of the DHEA-induced PCOS rodent model^42^. It is plausible that activation of ER stress in granulosa cells of PCOS patients and mice is independent of obesity.

The mRNA expression of TGF-β1 and CTGF, a downstream factor of TGF-β1 that mediates the pro-fibrotic actions of TGF-β1, was increased in granulosa cells of PCOS patients compared with control patients and the histological examination revealed the increased TGF-β1 protein expression in granulosa cells of the ovaries of PCOS patients. Granulosa cells of PCOS mice also exhibited higher expression of TGF-β1 protein than did those of control mice. These findings are in agreement with the earlier studies, which showed an increased serum level and ovarian expression of TGF-β1 in PCOS patients and the DHEA-induced PCOS rodent model^43–46^. The results of a microarray study involving granulosa cells of PCOS patients further strengthened the involvement of dysregulated TGF-β1 in the pathogenesis of PCOS^47^. However, the mechanism regulating TGF-β1 expression in the ovary of PCOS patients remains unclear. Our results show that treatment with an ER stress inducer, tunicamycin or thapsigargin, upregulated the expression of TGF-β1 and the secretion of a bioactive form of TGF-β1, as well as the mRNA expression of CTGF. Pretreatment with an ER stress inhibitor, TUDCA, alleviated ER stress-stimulated TGF-β1 and CTGF expression. The results of the interference assay suggest that XBP1(S), a transcription factor induced by ER stress, partly mediates the upregulation of TGF-β1 expression induced by ER stress. Future investigations would uncover the molecular mechanisms in detail by which activated ER stress induces TGF-β1 expression in granulosa cells. Recent studies report the stimulatory role of ER stress in TGF-β1 expression in various cells such as hepatocytes and fibroblasts^24, 25, 48, 49^. Our results indicate that ER stress is activated in granulosa cells of PCOS patients and ER stress activation in granulosa cells increased TGF-β1 expression in these cells.

We then examined the *in vivo* effects of ER stress inhibitors on ovarian fibrosis in the PCOS mouse model to determine whether activated ER stress in granulosa cells plays a role in the pathogenesis of PCOS. Among the various cellular functions that UPR might affect, we focused on ovarian fibrosis because recent studies show the pro-fibrotic role of ER stress and UPR in tissue fibrosis^22, 23^. Our *in vitro* experiments also showed that ER stress induced the expression of pro-fibrotic growth factors, TGF-β1 and CTGF, in granulosa cells, which play crucial roles in the regulation of extracellular matrix remodeling in human ovarian tissue^11–14^. Moreover, abnormalities in extracellular matrix remodeling, in part, account for the pathogenesis of PCOS^10^. Our results show that the ovaries of PCOS patients and mice showed an increase in fibrotic tissue in the interstitial area, with higher levels of collagen type I in the interstitial area and collagen type IV in the basement membrane of PCOS mice, consistent with earlier studies^10, 46, 50^^,^. Our results also show that treatment with an ER stress inhibitor, TUDCA or BGP-15, attenuated the increase in the area of fibrotic tissue and the deposition of collagen type I and IV observed in PCOS mice, with a concomitant reduction in TGF-β1 expression in granulosa cells. Taken together with our *in vitro* studies, which showed that ER stress inducers increased TGF-β1 expression in granulosa cells, the observations in the PCOS mouse model further suggest that ER stress activation in granulosa cells of PCOS patients induces ovarian fibrosis by upregulating TGF-β1 expression in granulosa cells. A similar mechanism is involved in the pathogenesis of liver fibrosis in HCV hepatitis or in genetic liver disease^24, 25^. In addition, recent studies uncovered the other three types of mechanisms by which ER stress induces tissue fibrosis. The first one involves the direct stimulatory effects of ER stress on fibroblasts to produce TGF-β1^48, 49^. The second one involves the role of ER stress in the induction of inflammation and apoptosis of epithelial cells, leading to epithelial cell loss, fibroblast activation, and, eventually, tissue fibrosis, a mechanism that is well-characterized in pulmonary and kidney fibrosis and inflammatory bowel diseases^51–53^. The third one involves the role of ER stress in the induction of the epithelial-mesenchymal transition, which is also observed in alveolar epithelial cells and renal tubular cells^54, 55^. Thus, ER stress plays a pro-fibrotic role in various ways, depending on the affected cell types or pathology.

TUDCA and BGP-15 administration did not improve the estrous cyclicity and the cystic follicle counts of PCOS mice. It might be attributed to a limitation of the present study utilizing a rodent PCOS model for *in vivo* experiments. None of the currently available PCOS models exhibit the complete range of abnormalities observed in PCOS patients and most of the PCOS models are androgen-induced^2^. The DHEA-induced model utilized in this study provides relevant information about the genesis of ovarian abnormalities, but the metabolic dysfunction is not completely phenotyped^34^. An example which indicates the imperfection of this model is that a report shows that even the 20-day treatment of DHEA-induced PCOS mice with metformin, the efficacy of which is well-recognized for PCOS treatment, exerted no effects on cyclicity and cystic follicle counts^56^. It remains unclear whether TUDCA and BGP-15 exert their therapeutic effects only on production of pro-fibrotic growth factors in granulosa cells and ovarian fibrosis, or also on other factors in PCOS pathology including disordered gonadotropin secretion, androgen excess, insulin resistance, ovarian dysfunction, and follicular arrest. Future studies with a novel PCOS animal model that spontaneously develops PCOS phenotype, without induction by steroids, would be required. Alternatively, the studies in clinical settings would also be useful since both TUDCA and BGP-15 are safe in humans^30, 32^. Another potential limitation of the present study is to utilize human granulosa-lutein cells for *in vitro* experiments. Given that intraovarian factors contribute to the impaired folliculogenesis and steroidogenesis in PCOS, human follicle culture which could recapitulate the *in vivo* environment would be more suitable for studying ovarian abnormalities in PCOS^2^.

In summary, we demonstrated that ER stress is activated in granulosa cells of the PCOS ovary. ER stress in granulosa cells induces TGF-β1 expression in these cells and contributes to ovarian fibrosis in the PCOS ovary. In addition to the role of ER stress as a pro-fibrotic stimulus, future studies might unravel additional mechanisms that explain how activated ER stress contributes to the pathogenesis of PCOS. Our findings suggest that ER stress is a potential therapeutic target in PCOS.

## Materials and Methods

### Human specimens

Granulosa-lutein cells and follicular fluid were aspirated from patients undergoing oocyte retrieval for IVF at the University of Tokyo Hospital and Matsumoto Ladies Clinic. The mRNA expression levels of various UPR genes, as well as TGF-β1 and CTGF, were examined in granulosa cells of 11 PCOS patients and ten control patients. Women with PCOS were diagnosed according to Rotterdam criteria^57^. The 11 patients with PCOS presented with oligo- or amenorrhea and a polycystic ovarian morphology. LH, FSH, E2, and PRL levels were measured between days 3 and 5 of the menstrual period. The AMH level was also measured. The inclusion criteria for patients without PCOS were a normal ovulatory cycle, no endocrine abnormalities, and a normal ovarian morphology by ultrasound.

Immunohistochemical analysis were performed on the ovaries of 3 PCOS and 3 control patients. Normal ovaries were obtained from women with regular menstrual cycles without hormonal treatment, who underwent radical or extended hysterectomy for carcinoma of the uterine cervix or endometrium. PCOS ovaries were obtained from women with oligo- or anovulation, who had undergone laparotomy and the findings of polycystic ovaries were histologically confirmed.

All experimental procedures were approved by The University of Tokyo review board, and the protocol for the experiments using human ovarian sections was approved by University of California San Diego review board, and signed informed consent was obtained from each patient. All methods were performed in accordance with the guidelines and regulations of the review board at The University of Tokyo and University of California San Diego, which are based on the ethical standards laid down in the 1964 declaration of Helsinki and its later amendments.

### PCOS animal model

A well-established PCOS mouse model was used in this study^34–36^. Three-week-old female Balb/C mice were obtained from Japan SLC, Inc. (Hamamatsu, Japan). To examine the activation of ER stress in the ovaries of PCOS mice, the animals were divided into two groups. The control group (n = 5) was s.c. injected daily with sesame oil for 20 days, and the PCOS group (n = 5) was s.c. injected daily with DHEA, (6 mg/100 g of body weight; Sigma-Aldrich, St. Louis, MO, USA) for 20 days. To examine the effects of ER stress inhibitors on fibrosis in the PCOS model, TUDCA or BGP-15 was utilized. Mice were divided into four groups. The control group (n = 5) was s.c. injected daily with sesame oil, followed by the oral administration of saline for 20 days. The PCOS group (n = 5) was s.c. injected daily with DHEA (6 mg/100 g of body weight), followed by the oral administration of saline for 20 days. The PCOS + TUDCA group (n = 5) was s.c. injected with DHEA, followed by the oral administration of TUDCA (50 mg/100 g of body weight; Tokyo Chemical Industry Co., Tokyo, Japan) for 20 days. The PCOS + BGP-15 group (n = 5) was s.c. injected with DHEA, followed by the oral administration of BGP-15 (Sigma-Aldrich, 3 mg/100 g of body weight) for 20 days. We decided the treatment doses of TUDCA and BGP-15 according to earlier studies^32, 33, 58^. The estrous cycle was timed between days 15 and 21 by analyzing cells in vaginal smears as previously reported^36^. The ovaries and serum were collected on day 21.

All experimental protocols were approved by the University of Tokyo Committee on the Use and Care of Animals. All methods were performed in accordance with the guidelines and regulations of the University of Tokyo Committee on the Use and Care of Animals.

### Isolation and culture of human granulosa-lutein cells

Isolation and culture of granulosa-lutein cells was performed as previously reported^28, 29^. All granulosa-lutein cells were cultured for 3–5 days prior to treatment.

### Treatment of human granulosa-lutein cells

To examine the effects of ER stress on TGF-β1 expression, granulosa-lutein cells were pre-incubated with the ER stress inhibitor TUDCA at 1 mg/mL for 24 h, followed by incubation with an ER stress inducer, 2.5 μg/mL tunicamycin (WAKO, Osaka, Japan) or 0.5 μM thapsigargin (Sigma-Aldrich), for 24 h. The optimal concentrations for the ER stress inhibitor and the ER stress inducers were determined according to our previous studies^28, 29^. To knock down XBP1(S), siRNA was obtained as the ON-TARGET plus SMART pool human XBP-1 siRNA (L-009552-00-020) from Dharmacon (GE Healthcare, Buckinghamshire, United Kingdom). The non-targeting siRNA control, ON-TARGET plus non-targeting pool (D-001810-10-20), was also obtained from Dharmacon. Granulosa-lutein cells were transfected with 50 nM siRNA for 24 h in Opti-MEM (Invitrogen, Carlsbad, CA, USA) using Lipofectamine RNAiMAX (Invitrogen). After transfection, the medium was removed, and the granulosa-lutein cells were treated with tunicamycin (2.5 μg/mL) for 24 h.

### RNA extraction, RT, and quantitative real-time PCR

Total RNA was extracted from ovaries of mice using ISOGEN (NIPPON GENE, Tokyo, Japan). One microgram of total RNA was reverse transcribed using the ReverTra Ace qPCR RT Master Mix with genomic DNA remover (TOYOBO, Osaka, Japan) in a volume of 40 μL. The cDNA template was synthesized from human granulosa-lutein cells using the SuperPrep Cell Lysis & RT Kit for qPCR (TOYOBO). To measure mRNA levels, quantitative real-time PCR was performed using the Light Cycler System (Roche Diagnostics GmBH, Mannheim, Germany). The UPR genes, namely, XBP1(S), HSPA5, ATF4, ATF6, and CHOP, were examined as markers of ER stress activation. The mRNA expression levels were normalized to that of glyceraldehyde-3-phosphate dehydrogenase (GAPDH), which served as an internal control. The primer sequences were shown in Supplementary Table 2. Except for human XBP1(S), the PCR conditions were as follows: 40 cycles at 98 °C for 10 sec, 60 °C for 10 sec, and 68 °C for 30 sec. The PCR conditions for human XBP1(S) were as follows: 40 cycles at 98 °C for 10 sec, 66 °C for 10 sec, and 68 °C for 30 sec. All samples were analyzed in triplicate or quadruplicate for *in vitro* experiments.

### Histology

Human and mouse ovaries were fixed in 10% neutral-buffered formalin, embedded in paraffin, and sectioned at a thickness of 3 μm. Sections obtained from the center of each ovary were stained with hematoxylin and eosin or Masson’s trichrome stain.

### Immunohistochemistry

The sections were immunostained with an anti-DDIT3 (9C8) antibody (1:200, Abcam, Tokyo, Japan), anti-IRE1 (phospho S724) antibody (1:500, Abcam), anti-PERK (phospho T980 or phospho T982) antibody (1:500, Abcam), or anti-TGF-β1 antibody (1:1000, Abcam) using the EnVision + Dual Link System/HRP (DAB) Kit (Dako, Tokyo, Japan). Isotype-specific IgG served as the negative control. Antigen retrieval was performed using target retrieval solution (Dako). Immunohistochemistry was performed three independent times using identical samples.

### *In situ* hybridization

*In situ* hybridization was performed using the ISHR Starting Kit (Nippon Gene, Toyama, Japan) as previously reported^27^. Antisense and sense digoxigenin (DIG)-labeled RNA probes for XBP1(S) and HSPA5 were synthesized from mouse cDNA templates sub-cloned into the PCR-2 TOPO vector (Invitrogen). The primer sequences used for RT-PCR were as follows: XBP1(S) (sense, 5′-GCTGAGTCCGCAGCAGGTGC-3′; antisense, 5′-CATGACAGGGTCCAACTTGTCCAG-3′) and HSPA5 (sense, 5′-GACATTTGCCCCAGAAGAAA-3′; antisense, 5′-CTCATGACATTCAGTCCAGCA-3′). Vectors linearized with an appropriate restriction enzyme served as templates for the synthesis of RNA probes using SP6 or T7 RNA polymerase. Hybridization with a sense probe served as the control. *In situ* hybridization was performed three independent times using identical samples.

### Quantitative analysis of immunohistochemistry and *in situ* hybridization

For quantitative analysis, the images were analyzed using Image J software program (National Institute of Health, Bethesda, MD, USA)^59^. For phospho-PERK, phospho-IRE1, CHOP, TGF-β1, XBP1(S), and HSPA5, the number of positively stained granulosa cells of the five randomly-selected follicles were counted in the ovaries from 3 different patients or 5 different mice. For Masson’s trichrome staining and immunohistochemical staining for collagen type I and type IV, stained area was evaluated in three randomly-selected fields of the ovaries from 3 different patients or 5 different mice.

### TGF-β1 assay

The human bioactive TGF-β1 concentration in cell culture supernatants was measured using the Quantikine ELISA Kit (R&D Systems, Minneapolis, MN, USA). To activate latent TGF-β1, cell culture supernatants were acidified with 100 μL of 1 M HCl and incubated at room temperature for 10 min. The samples were then neutralized with 100 μL of 0.5 M HEPES/1.2 M NaOH. The absorbance was measured at 450 nm using an Epoch Multi-Volume Spectrophotometer (BioTek Instruments, Winooski, VT, USA). The limit of sensitivity was 15.4 pg/mL. All samples were analyzed in sextuplicate.

### Testosterone assay

The mouse serum testosterone concentration was measured using the Testosterone ELISA Kit (Enzo Life Sciences, Farmingdale, NY, USA). The absorbance was measured at 450 nm using an Epoch Multi-Volume Spectrophotometer. The limit of sensitivity was 5.67 pg/mL.

### Statistical analysis

Statistical analyses were performed using JMP Pro 11 software (SAS Institute Inc., Cary, NC, USA). All results are shown as means ± SEM. Data were analyzed using the Wilcoxon rank sum test for paired comparisons, and the Tukey–Kramer HSD test for multiple comparisons. A *p*-value less than 0.05 was considered significant.

## Electronic supplementary material

Supplementary information

## References

[1] Bozdag G, Mumusoglu S, Zengin D, Karabulut E, Yildiz BO (2016). The prevalence and phenotypic features of polycystic ovary syndrome: a systematic review and meta-analysis. Hum Reprod..

[2] Dumesic DA (2015). Scientific Statement on the Diagnostic Criteria, Epidemiology, Pathophysiology, and Molecular Genetics of Polycystic Ovary Syndrome. Endocr Rev..

[3] Ma X (2007). Proteomic analysis of human ovaries from normal and polycystic ovarian syndrome. Mol Hum Reprod..

[4] Schmidt J (2014). Differential expression of inflammation-related genes in the ovarian stroma and granulosa cells of PCOS women. Molr Human Reprod..

[5] Zhao Y (2015). Up-regulated expression of WNT5a increases inflammation and oxidative stress via PI3K/AKT/NF-kappaB signaling in the granulosa cells of PCOS patients. J Clin Endocrinol Metab..

[6] Adams J (2016). Enhanced Inflammatory Transcriptome in the Granulosa Cells of Women With Polycystic Ovarian Syndrome. J Clin Endocrinol Metab..

[7] Chattopadhayay R (2010). Effect of follicular fluid oxidative stress on meiotic spindle formation in infertile women with polycystic ovarian syndrome. Gynecol Obstet Invest..

[8] Karuputhula NB, Chattopadhyay R, Chakravarty B, Chaudhury K (2013). Oxidative status in granulosa cells of infertile women undergoing IVF. Syst Biol Reprod Med..

[9] Gonzalez F, Rote NS, Minium J, Kirwan JP (2006). Reactive oxygen species-induced oxidative stress in the development of insulin resistance and hyperandrogenism in polycystic ovary syndrome. J Clin Endocrinol Metab..

[10] Hughesdon PE (1982). Morphology and morphogenesis of the Stein-Leventhal ovary and of so-called “hyperthecosis”. Obstet Gynecol Surv..

[11] Cheng JC, Chang HM, Fang L, Sun YP, Leung PC (2015). TGF-beta1 Up-Regulates Connective Tissue Growth Factor Expression in Human Granulosa Cells through Smad and ERK1/2 Signaling Pathways. PLoS One.

[12] Chang HM (2016). Connective tissue growth factor mediates growth differentiation factor 8-induced increase of lysyl oxidase activity in human granulosa-lutein cells. Mol Cell Endocrinol..

[13] Chang HM (2016). Activin A-induced increase in LOX activity in human granulosa-lutein cells is mediated by CTGF. Reproduction..

[14] Fang Y (2016). Transforming growth factor-beta1 increases lysyl oxidase expression by downregulating MIR29A in human granulosa lutein cells. Reproduction..

[15] Raja-Khan N, Urbanek M, Rodgers RJ, Legro RS (2014). The role of TGF-beta in polycystic ovary syndrome. Reprod Sci..

[16] Walter P, Ron D (2011). The unfolded protein response: from stress pathway to homeostatic regulation. Science..

[17] Ozcan L, Tabas I (2012). Role of endoplasmic reticulum stress in metabolic disease and other disorders. Annu Rev Med..

[18] Grootjans J, Kaser A, Kaufman RJ, Blumberg RS (2016). The unfolded protein response in immunity and inflammation. Nat Rev Immunol..

[19] Hasnain SZ, Lourie R, Das I, Chen AC, McGuckin MA (2012). The interplay between endoplasmic reticulum stress and inflammation. Immunol Cell Biol..

[20] Navid F, Colbert RA (2017). Causes and consequences of endoplasmic reticulum stress in rheumatic disease. Nat Rev Rheumatol..

[21] Ron D, Walter P (2007). Signal integration in the endoplasmic reticulum unfolded protein response. Nat Rev Mol Cell Biol..

[22] Lenna S, Trojanowska M (2012). The role of endoplasmic reticulum stress and the unfolded protein response in fibrosis. Curr Opin Rheumatol..

[23] Tanjore H, Lawson WE, Blackwell TS (2013). Endoplasmic reticulum stress as a pro-fibrotic stimulus. Biochim Biophys Acta..

[24] Chusri P (2016). HCV induces transforming growth factor beta1 through activation of endoplasmic reticulum stress and the unfolded protein response. Sci Rep..

[25] Mencin A (2007). Alpha-1 antitrypsin Z protein (PiZ) increases hepatic fibrosis in a murine model of cholestasis. Hepatology..

[26] Kassan M (2012). Endoplasmic reticulum stress is involved in cardiac damage and vascular endothelial dysfunction in hypertensive mice. Arterioscler Thromb Vasc Biol..

[27] Harada M (2015). Evidence of the activation of unfolded protein response in granulosa and cumulus cells during follicular growth and maturation. Gynecol Endocrinol..

[28] Takahashi N (2016). A potential role of endoplasmic reticulum stress in development of ovarian hyperstimulation syndrome. Mol Cell Endocrinol..

[29] Takahashi N (2017). A potential role for endoplasmic reticulum stress in progesterone deficiency in obese women. Endocrinology..

[30] Boatright JH, Nickerson JM, Moring AG, Pardue MT (2009). Bile acids in treatment of ocular disease. J Ocul Biol Dis Infor..

[31] Hetz C, Chevet E, Harding HP (2013). Targeting the unfolded protein response in disease. Nat Rev Drug Discov..

[32] Kennedy TL (2016). BGP-15 Improves Aspects of the Dystrophic Pathology in mdx and dko Mice with Differing Efficacies in Heart and Skeletal Muscle. Am J Pathol..

[33] Wu LL (2015). Mitochondrial dysfunction in oocytes of obese mothers: transmission to offspring and reversal by pharmacological endoplasmic reticulum stress inhibitors. Development..

[34] Elia E (2006). The mechanisms involved in the action of metformin in regulating ovarian function in hyperandrogenized mice. Mol Hum Reprod..

[35] Solano ME (2006). Metformin prevents embryonic resorption induced by hyperandrogenisation with dehydroepiandrosterone in mice. Reprod Fertil Dev..

[36] Lai H (2014). High-fat diet induces significant metabolic disorders in a mouse model of polycystic ovary syndrome. Biol Reprod..

[37] Meyerovich K, Ortis F, Allagnat F, Cardozo AK (2016). Endoplasmic reticulum stress and the unfolded protein response in pancreatic islet inflammation. J Mol Endocrinol..

[38] Rani V, Deep G, Singh RK, Palle K, Yadav UC (2016). Oxidative stress and metabolic disorders: Pathogenesis and therapeutic strategies. Life Sci..

[39] Hasnain SZ, Prins JB, McGuckin MA (2016). Oxidative and endoplasmic reticulum stress in beta-cell dysfunction in diabetes. J Mol Endocrinol..

[40] Gonzalez F (2012). Inflammation in Polycystic Ovary Syndrome: underpinning of insulin resistance and ovarian dysfunction. Steroids..

[41] Longo M (2016). Pathologic endoplasmic reticulum stress induced by glucotoxic insults inhibits adipocyte differentiation and induces an inflammatory phenotype. Biochim Biophys Acta..

[42] Maliqueo M, Benrick A, Stener-Victorin E (2014). Rodent models of polycystic ovary syndrome: phenotypic presentation, pathophysiology, and the effects of different interventions. Semin Reprod Med..

[43] Hatzirodos N (2011). Linkage of regulators of TGF-beta activity in the fetal ovary to polycystic ovary syndrome. FASEB J..

[44] Tal R, Seifer DB, Shohat-Tal A, Grazi RV, Malter HE (2013). Transforming growth factor-beta1 and its receptor soluble endoglin are altered in polycystic ovary syndrome during controlled ovarian stimulation. Fertil Steril..

[45] Miao ZL (2012). The intervention effect of Rosiglitozone in ovarian fibrosis of PCOS rats. Biomed Environ Sci..

[46] Zhang X (2013). Dehydroepiandrosterone induces ovarian and uterine hyperfibrosis in female rats. Hum Reprod..

[47] Kaur S (2012). Differential gene expression in granulosa cells from polycystic ovary syndrome patients with and without insulin resistance: identification of susceptibility gene sets through network analysis. J Clin Endocrinol Metab..

[48] Matsuzaki S (2015). Physiological ER Stress Mediates the Differentiation of Fibroblasts. PLoS One..

[49] Ranga Rao, S., Subbarayan, R., Ajitkumar, S., & Murugan Girija, D. 4PBA Strongly Attenuates Endoplasmic Reticulum Stress, Fibrosis and Mitochondrial Apoptosis Markers in Cyclosporine Treated Human Gingival Fibroblasts. *J Cell Physiol*. doi:10.1002/jcp.25836 (2017) [Epub ahead of print]. 10.1002/jcp.25836 28158898

[50] Bulut G, Kurdoglu Z, Donmez YB, Kurdoglu M, Erten R (2015). Effects of jnk inhibitor on inflammation and fibrosis in the ovary tissue of a rat model of polycystic ovary syndrome. Int J Clin Exp Ppathol..

[51] Tanjore H, Blackwell TS, Lawson WE (2012). Emerging evidence for endoplasmic reticulum stress in the pathogenesis of idiopathic pulmonary fibrosis. Am J Physiol Lung Cell Mol Physiol..

[52] Chiang CK (2011). Endoplasmic reticulum stress implicated in the development of renal fibrosis. Mol Med..

[53] Heazlewood CK (2008). Aberrant mucin assembly in mice causes endoplasmic reticulum stress and spontaneous inflammation resembling ulcerative colitis. PLoS Med..

[54] Tanjore H (2011). Alveolar epithelial cells undergo epithelial-to-mesenchymal transition in response to endoplasmic reticulum stress. J Biol Chem..

[55] Pallet N (2008). Cyclosporine-induced endoplasmic reticulum stress triggers tubular phenotypic changes and death. Am J Transplant..

[56] Huang Y (2015). Impaired oocyte quality induced by dehydroepiandrosterone is partially rescued by metformin treatment. PLoS One..

[57] Revised 2003 consensus on diagnostic criteria and long-term health risks related to polycystic ovary syndrome. *Fertil Steril*. **81**, 19-25 (2004). 10.1016/j.fertnstert.2003.10.004 14711538

[58] Dong Y (2010). Reduction of AMP-activated protein kinase alpha2 increases endoplasmic reticulum stress and atherosclerosis *in vivo*. Circulation..

[59] Schneider CA, Rasband WS, Eliceiri KW (2012). NIH Image to ImageJ: 25 years of image analysis. Nature Methods.

