# Supplementary material for: Activation of Endoplasmic Reticulum Stress in Granulosa Cells from Patients with Polycystic Ovary Syndrome Contributes to Ovarian Fibrosis
Source: Sci Rep. 2017 Sep 7;7:10824. doi: 10.1038/s41598-017-11252-7 (PMC5589802; doi:10.1038/s41598-017-11252-7)
Supplement: Supplementary file 1 — Supplementary information [file 41598_2017_11252_MOESM1_ESM.pdf]

Supplementary Information

Activation of Endoplasmic Reticulum Stress in Granulosa Cells from Patients with Polycystic Ovary Syndrome Contributes to Ovarian Fibrosis

Nozomi Takahashi, Miyuki Harada*, Yasushi Hirota, Emi Nose, Jerilee MK Azhary, Hiroshi Koike, Chisato Kunitomi, Osamu Yoshino, Gentaro Izumi, Tetsuya Hirata, Kaori Koga, Osamu Wada-Hiraike, R. Jeffrey Chang, Shunichi Shimasaki, Tomoyuki Fujii, Yutaka Osuga

Supplementary Figure 1. Comparison of characteristics between control and PCOS mice. Three-week-old female mice were divided into two groups. The control group (N=5) was s.c. injected daily with sesame oil for 20 days. The PCOS group (N=5) was s.c. injected daily with DHEA (6 mg/100 g of body weight) for 20 days. The ovaries and serum were collected on day 21. (A) The serum testosterone concentration was measured by ELISA. (B, C) Cross-sections of ovaries were stained with hematoxylin and eosin. The number of cystic follicles was determined from the center of each ovary (B). Representative ovarian morphology from control and PCOS mice is shown (C). (D) Representative estrous cycles from control and PCOS mice are shown. The estrous cycle was timed between days 15 and 21 by analyzing cells in vaginal smears. The values represent means ± SEM. *, $p < 0.05$. D, diestrous; E, estrous; M, metaestrous; P, proestrous.

Supplementary Figure 1, Takahashi N. et al.

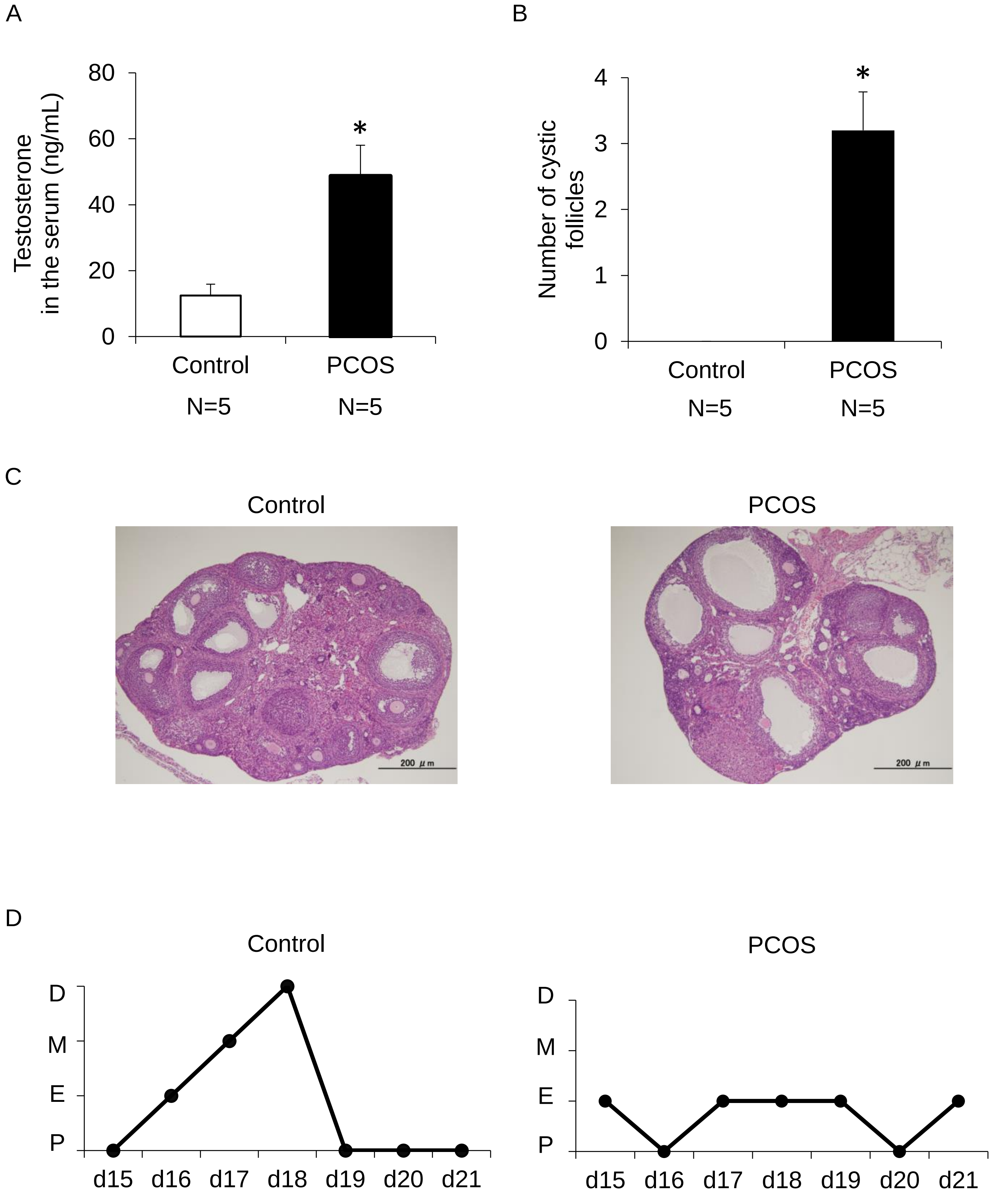

Supplementary Figure 2. Effect of thapsigargin on TGF-β1 mRNA expression levels by cultured human granulosa-lutein cells.

(A-D), Granulosa-lutein cells were incubated with thapsigargin at 0.5 μM for 0, 3, 9, or 24 h. (E-H) Granulosa-lutein cells were pre-incubated with TUDCA at 1 mg/mL for 24 h, followed by treatment with thapsigargin at 0.5 μM for 24 h. TGF-β1 , XBP1(S), HSPA5, and CHOP mRNA expression levels in granulosa-lutein cells were measured by real-time PCR and normalized to that of GAPDH. The values represent means ± SEM of triplicate or quadruplicate experiments, relative to the mean control value. The results are representative of at least three independent experiments using three different samples. The letters denote significant difference. *, $p < 0.05$. Tg, thapsigargin; TUDCA, tauroursodeoxycholic acid.

Supplementary Figure 2, Takahashi N. et al.

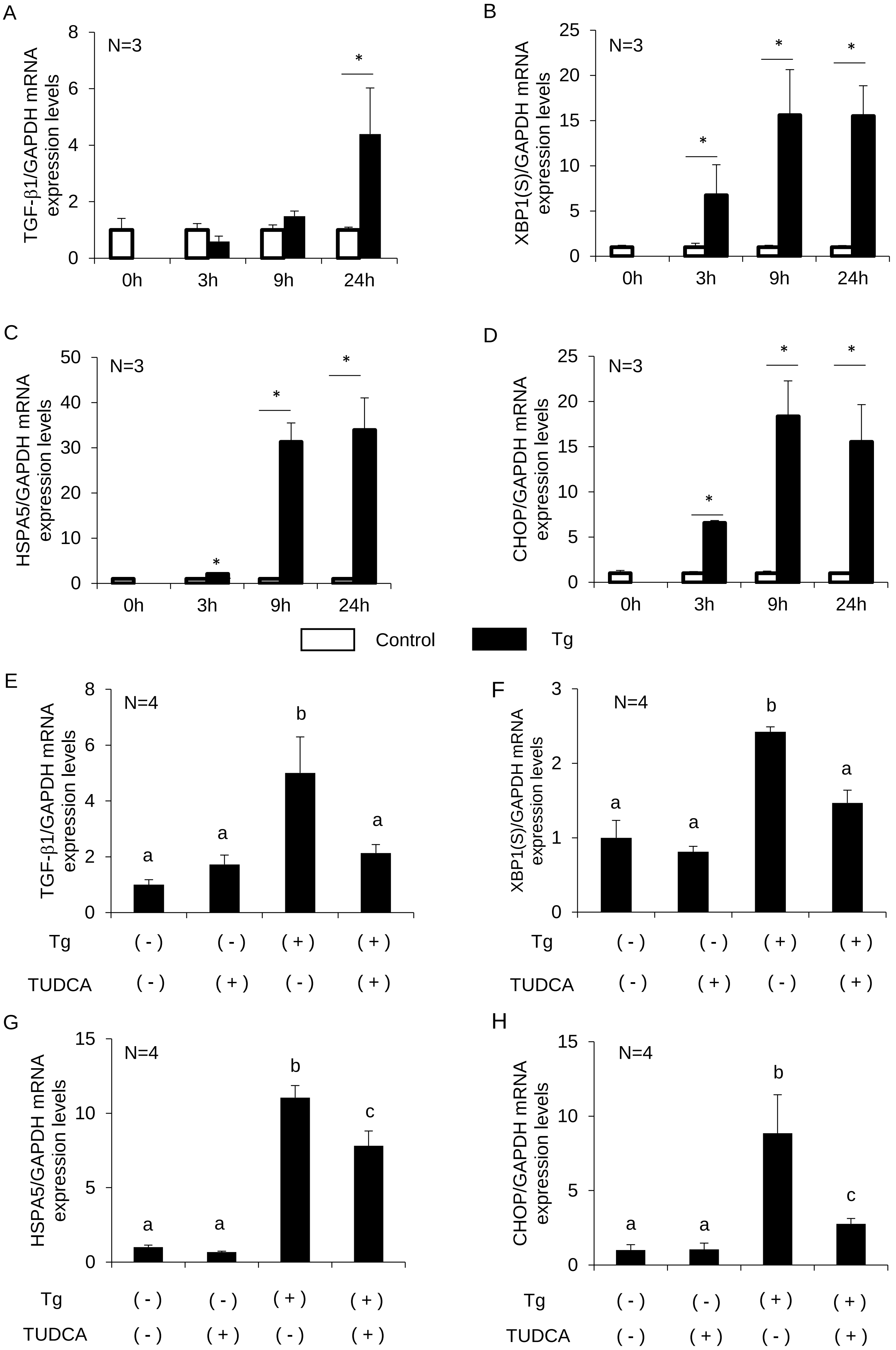

Supplementary Figure 3. Comparison of characteristics between control, PCOS, PCOS + TUDCA, PCOS + BGP-15 mice.

Three-week-old female mice were divided into four groups. The control group (N=5) was s.c. injected daily with sesame oil, followed by the oral administration of saline for 20 days. The PCOS group (N=5) was s.c. injected daily with DHEA (6 mg/100 g of body weight), followed by the oral administration of saline for 20 days. The PCOS + TUDCA group (N=5) was s.c. injected daily with DHEA, followed by the oral administration of TUDCA (50 mg/100 g of body weight) for 20 days. The PCOS + BGP-15 (N=5) group was s.c. injected daily with DHEA, followed by the oral administration of BGP-15 (3 mg/100 g of body weight) for 20 days. The ovaries were collected on day 21. (A) Cross-sections of ovaries were stained with hematoxylin and eosin and the number of cystic follicles was determined from the center of each ovary. (B) Representative estrous cycles from control, PCOS, PCOS + TUDCA, and PCOS + BGP-15 mice are shown. The estrous cycle was timed between days 15 and 21 by analyzing cells in vaginal smears. The letters denote significant differences between groups. TUDCA, tauroursodeoxycholic acid; D, diestrous; E, estrous; M, metaestrous; P, proestrous.

Supplementary Figure 3, Takahashi N. et al.

A

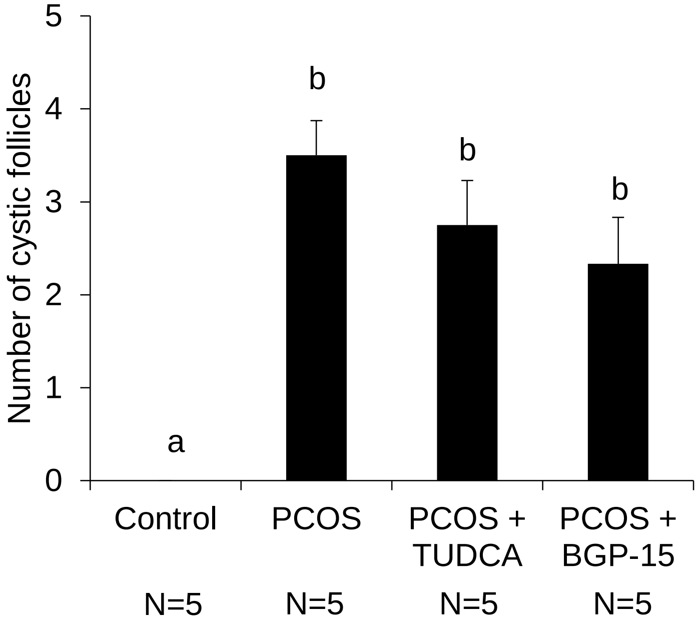

B

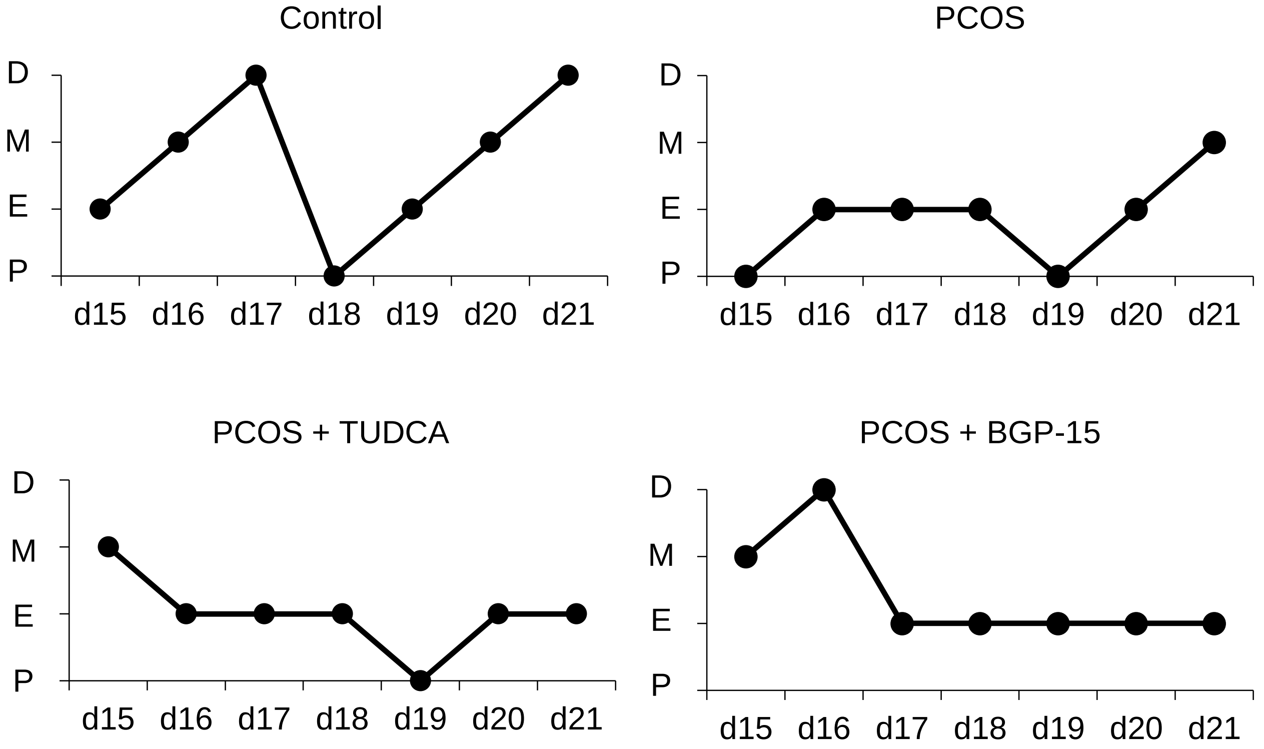

Supplementary Table 1, Takahashi N. et al.

Comparison of characteristics between control and PCOS patients

	**Control (N=10)**	**PCOS (N=11)**	***p*-value**
Age (year)	36 (34-43)	34 (29-41)	0.0661
BMI (kg/m^2)	19.3 (18.6-21.2)	19.3 (16.7-20.9)	0.7237
Number of retrieved oocytes	7 (5-12)	12 (3-21)	0.0828
LH (mIU/mL)	3.85 (2.3-6.5)	9.9 (2.2-20.6)	0.0002
FSH (mIU/mL)	8.55 (4.4-16.3)	6.4 (4.3-10)	0.1415
LH/FSH	0.45 (0.34-0.92)	1.32 (0.43-3.38)	0.0002
E2 (pg/mL)	29.7 (18.0-45.7)	39.7 (16.0-68.7)	0.1547
PRL (ng/mL)	10.0 (7.3-26.6)	10.0 (5.0-41.1)	0.5669
AMH (ng/mL)	2.56 (0.1-3.0)	6.32 (3.7-17.0)	0.0002

Median (range)
BMI: body mass index, LH: luteinizing hormone,
FSH: follicle stimulating hormone, E2: estradiol, PRL: prolactin,
AMH: anti-Müllerian hormone

Supplementary Table 2, Takahashi N. et al.

List of primers used for real-time PCR.

Gene Name	Forward Primer 5′- 3′	Reverse Primer 5′- 3′
human XBP1(S)	TGCTGAGTCCGCAGCAGGTG	GCTGGCAGGCTCTGGGGAAG
human HSPA5	CGAGGAGGAGGACAAGAAGG	CGAGGAGGAGGACAAGAAGG
human ATF4	GGCTGGCTGTGGATGGGTTG	CTCCTGGACTAGGGGGGCAA
human ATF6	TCAGACAGTACCAACGCTTATGC	GTTGTACCACAGTAGGCTGAGA
human CHOP	GGAGAACCAGGAAACGGAAAC	TCTCCTTCATGCGCTGCTTT
human TGF-β1	GGCCAGATCCTGTCCAAGC	GTGGGTTTCCACCATTAGCAC
human CTGF	CAGCATGGACGTTCGTCTG	AACCACGGTTTGGTCCTTGG
human GAPDH	GGACCTGACCTGCCGTCTA	CTGCTTCACCACCTTCTTGA
mouse XBP1(S)	GCTGAGTCCGCAGCAGGTGC	CATGACAGGGTCCAACTTGTCCA G
mouse TGF-β1	CCACCTGCAAGACCATCGAC	CTGGCGAGCCTTAGTTTGGAC
mouse CTGF	GGCCTCTTCTGCGATTTCG	GCAGCTTGACCCTTCTCGG
mouse GAPDH	AGGTCGGTGTGAACGGATTTG	TGTAGACCATGTAGTTGAGGTCA
